# Genomic association for sexual precocity in beef heifers using pre-selection of genes and haplotype reconstruction

**DOI:** 10.1371/journal.pone.0190197

**Published:** 2018-01-02

**Authors:** Luciana Takada, Marina M. D. Barbero, Henrique N. Oliveira, Gregório M. F. de Camargo, Gerardo A. Fernandes Júnior, Rusbel R. Aspilcueta-Borquis, Fabio R. P. Souza, Arione A. Boligon, Thaise P. Melo, Inaê C. Regatieri, Fabieli L. B. Feitosa, Larissa F. S. Fonseca, Ana F. B. Magalhães, Raphael B. Costa, Lucia G. Albuquerque

**Affiliations:** Departamento de Zootecnia—São Paulo State University—UNESP, Jaboticabal, São Paulo, Brazil; Faculty of Animal Sciences and Food Engineering, University of São Paulo, BRAZIL

## Abstract

Reproductive traits are of the utmost importance for any livestock farming, but are difficult to measure and to interpret since they are influenced by various factors. The objective of this study was to detect associations between known polymorphisms in candidate genes related to sexual precocity in Nellore heifers, which could be used in breeding programs. Records of 1,689 precocious and non-precocious heifers from farms participating in the Conexão Delta G breeding program were analyzed. A subset of single nucleotide polymorphisms (SNP) located in the region of the candidate genes at a distance of up to 5 kb from the boundaries of each gene, were selected from the panel of 777,000 SNPs of the High-Density Bovine SNP BeadChip. Linear mixed models were used for statistical analysis of early heifer pregnancy, relating the trait with isolated SNPs or with haplotype groups. The model included the contemporary group (year and month of birth) as fixed effect and parent of the animal (sire effect) as random effect. The fastPHASE® and GenomeStudio® were used for reconstruction of the haplotypes and for analysis of linkage disequilibrium based on r^2^ statistics. A total of 125 candidate genes and 2,024 SNPs forming haplotypes were analyzed. Statistical analysis after Bonferroni correction showed that nine haplotypes exerted a significant effect (p<0.05) on sexual precocity. Four of these haplotypes were located in the Pregnancy-associated plasma protein-A2 gene (*PAPP-A2)*, two in the Estrogen-related receptor gamma gene *(ESRRG)*, and one each in the Pregnancy-associated plasma protein-A gene *(PAPP-A)*, Kell blood group complex subunit-related family *(XKR4)* and *mannose-binding lectin genes (MBL-1)* genes. Although the present results indicate that the *PAPP-A2*, *PAPP-A*, *XKR4*, *MBL-1* and *ESRRG* genes influence sexual precocity in Nellore heifers, further studies are needed to evaluate their possible use in breeding programs.

## Introduction

Brazil currently has the largest commercial beef cattle herd in the world, with about 215.2 million heads, and is the world’s second-largest producer and the largest exporter of beef [1]. The analysis of beef cattle production systems shows that reproductive traits are of economic importance for profitability. In a study on Nellore cattle in Brazil, Brumatti et al., [2] concluded that reproductive traits (sexual precocity and stayability in the herd) are 4 to 13 times more important than carcass and growth traits. Pravia et al., [3] reported a three times greater importance of reproductive traits compared to traits related to growth and feed intake in a production system of Hereford cattle in Uruguay. The authors therefore recommend reproductive traits to be a selection objective for producers that wish to increase the profitability of their herds.

Sexual precocity and longevity of females in the herd show a high and positive genetic correlation [4, 5, 6], indicating that selection for more precocious animals concomitantly selects for animals that remain cycling in the herd for a longer period. The cost of fixed capital formation of the dam is thus diluted since the animal remains in the herd for a larger number of productive cycles. This is also observed phenotypically since precocious heifers are more likely to stay in the herd than non-precocious heifers [7].

The use of quantitative and molecular genetics permits the identification and validation of SNPs that influence phenotypes, as well as their subsequent utilization as markers to select traits of interest [8]. Using SNPs located close to genetic markers previously associated with reproductive traits, Cochran et al., [9] found 40 SNPs associated with pregnancy rate (p<0.05), with the genes being related to the endocrine system, cell signaling, immune system, and inhibition of apoptosis. The knowledge of genes that influence reproductive traits in Nellore cattle, as well as of the contribution of each gene, is important for understanding the biological mechanism that influences the expression of a phenotype and for future genetic evaluation.

Although the hypothalamic-pituitary-gonadal axis and related genes are important for the onset of puberty, it is unlikely that puberty can be explained by only a small number of genes associated with a single trait. After all, puberty is a complex phenotype that is influenced by many other processes, including those involved in energy balance and brain development [10]. Therefore, the general objective of this study was to detect polymorphisms associated with sexual precocity in Nellore heifers using a method that combines the pre-selection of candidate genes (genes related to folliculogenesis and gonads, follicular maturation, hypothalamus, pituitary, pregnancy, and puberty) and the reconstruction of haplotypes.

## Material and methods

### Animals and sample collection

This study was approved by the Ethics Committee on Animal Experimentation (CEUA) of the School of Agricultural and Veterinarian Sciences (FCAV/UNESP) Jaboticabal—São Paulo Brazil (protocol nº17958/15).

Data from 1,689 precocious and non-precocious Nellore heifers were available for breeding with sires at about 16 months of age in herds participating in the Conexão Delta G breeding program were used. All heifers of these herds are mated for the first time at an age close to 16 months, irrespective of weight and body conditions, and continue in the breeding season for about 3 months. During this period, heifers are subjected to multiple-sire natural mating, with a sire-cow ratio of 1:50. Approximately 60 days after the end of the breeding season pregnancy of the heifers is confirmed by rectal palpation. In the herds studied, 25.82% of the heifers became pregnant in this first breeding season.

The biological material was collected in previous performed studies by Costa et al. [11] and Irano et al. [12]. Hair follicles were collected from the brush of the tails (about 100 follicles/animal) during the breeding season of the heifers, and were stored in envelopes identified with the number of the animal, year of birth and breeding season. The follicles were carefully stored at 4°C until the time of DNA extraction.

### DNA extraction and quantification

About 40 hair follicles/animal were used. The follicles were cut with clean flame-sterilized scissors so that only the bulbs remained in the 1.5-ml microcentrifuge tubes. DNA was extracted from these hair follicles using the NucleoSpin Tissue Kit (Macherey-Nagel^®^) according to manufacturer instructions. A Nanodrop 1000 spectrophotometer (ThermoScientific, USA, 2008) was used to verify the quantity and quality of the extracted DNA. The DNA extracted from the hair follicles was of great quality, its concentration was higher than 100 ng/μL in 97% of, and the samples the 260:280 ratio ranged from 1.8 to 2.0, allowing use of a good material for the genotyping.

### Candidate genes

The candidate genes included 125 genes encoding proteins that are somehow related to age at first calving. All genes were chosen based on studies indicating their participation in folliculogenesis and gonad development (*ACVR*, *STAR*, *HSD17B3*), oocyte maturation (*BMP15*, *GDF9*) [9], hypothalamus and Central nervous system (*GnRH*, *GPR 54*, *KISS-1*, *XKR4*), pituitary (*ACTH*, *PRL*, *LHR*, *FSHR)* or pregnancy (*IFN-τ*, *MBL-1*, *PAPPA-A*, *PAPPA2*), or are genes that have been associated with puberty in previous studies (*PPARG*, *ESRRG*, *PROP1*) [10]. The complete list of the candidate genes is shown in “S1 Table”.

### SNP panel and genotyping

For the study of candidate genes, SNPs of the panel of 777,000 SNPs of the Illumina High-Density Bovine BeadChip located within or close to these genes were analyzed. The SNPs were chosen based on the position of the gene in the genome (GenBank) and the position of the SNP in the Illumina panel. All SNPs located up to 5 kilobases (kb) from the proposed genes were selected. This chip offers uniform coverage across the genome of taurine breeds, with an average spacing between markers of 5 kb, in addition to a minor allele frequency (MAF) of 0.25 (Illumina® SNP Genotyping).

Genotyping was performed following to the Infinium ® II Assay Multi-Sample protocol in a HiScan™ SQ System (Illumina). A total of 1.689 precocious and non-precocious heifers were genotyped. The GenomeStudio® program was applied to ensure the quality of the samples and remove samples with a call rate less than 90%. Based on this criterion 20 samples were excluded. SNPs that exhibited significant deviation from Hardy-Weinberg equilibrium (p<0.00001), which indicates genotyping errors, were removed. SNPs with an MAF < 0.01 and a call frequency < 0.9 were also eliminated. To generate the files with the information of each SNP for each animal, the same program was used according to the Illumina "TOP / BOT" designation.

### Haplotype reconstruction

After selection of the SNPs within the region of the candidate genes, the fastPHASE® program [13] was used for reconstruction of the haplotypes. The results were entered into the Haploview v4.1 program [14] to estimate the linkage disequilibrium between SNP pairs based on r^2^ statistics [15] and to obtain the allele patterns formed by each haplotype. SNPs that were not part of any haplotype were considered isolated markers.

### Statistical analysis

The trait evaluated was early heifer pregnancy. This is considered a binary trait in which value 1 is attributed to heifers with a positive diagnosis of pregnancy at 16 months and value 0 to those with a negative diagnosis. Although the trait has a binary distribution, linear models were used for this analysis because of their easier execution and interpretation of the results. Thus, the association with the trait of interest was verified using a linear mixed model, for each isolated or haplotype SNP.

It was used for all markers, the model included the contemporary group as fixed effect and the parent of the animal (sire effect) as random effect. The contemporary group was defined by concatenation of farm, year and month of birth of the animal. An identification code of the father (ghost sires) was generated for each animal with unknown sire to allow analysis with the software chosen.

To define the blocks of SNPs that form the haplotypes, was using the algorithm of Gabriel et al. [16], which considers the confidence interval D ', statistical implemented in Haploview software. Regarding the analysis of isolated SNPs, the copy number of the most frequent allele present in the genotype of the animal was included as a covariate. Where it was possible to detect the presence of haplotypes in the segments, the analyses were performed considering the block of haplotype-forming SNPs. In addition, in the latter case variables containing the copy number of each allele detected in the population, except for the least occurrence, were included as covariates. The model used can be written as:

$$y_{ijk}=CG_{i}+s_{j}+\sum_{l=1}^{\mathrm{n-}1}b_{l}x_{\mathrm{ijkl}}+e_{ijk}$$

where *y*_ijk_ is the observed value of the trait studied for animal *k* of sire *j* in contemporary group *i*; *CG*_i_ is the fixed effect of contemporary group *i*; *s*_j_ is the random effect of sire *j*; *b*_l_ is the regression coefficient of the copy number of allele *l* on the trait studied; *x*_ijkl_ is the copy number of allele *l* in the animal; *e*_ijk_ is the random error, and *n* is the number of alleles for the marker analyzed.

The Mixed Procedure of the SAS® statistical package was used in all analyzes. The Bonferroni method correction for multiple comparisons was applied to ensure maintenance of the error rate of the statistical tests. For each polymorphism, the allele and genotype frequencies were calculated according to [17].

## Results

It ranged from 0 to 199 the number of SNPs of the High DensityBovine SNP BeadChip found within each region of the candidate genes (“S1 Table”). No SNPs were detected for 11 of the candidate genes, which could therefore not be analyzed. Thus, the remaining 114 candidate genes and haplotype blocks were assembled and evaluated regarding their effects on early pregnancy. The haplotype blocks were formed by 2 to 20 SNPs.

S2 Table. The S2 Table shows the location of significant haplotypes and the position of the SNPs in the *PAPP-A2*, *PAPP-A*, *MBL-1*, *ESRRG and XKR4* genes.

For analysis 2024 SNPs were used. Statistical analysis of the 114 genes revealed a significant effect (p<0.05) of the nine haplotype blocks located in the *PAPP-A2*, *PAPP-A*, *MBL-1*, *ESRRG* and *XKR4* genes on early heifer pregnancy. In the *PAPPA-A2* gene, four haplotypes formed by 20, 18, 5 and 5 SNPs exerted significant effects (p<0.05) (Table 1). One haplotype with 2 SNPs was found in the *PAPPA-A* gene (Table 2), one haplotype with 4 SNPs in the *XKR4* gene (Table 3), one haplotype with 2 SNPs in the *MBL-1* gene (Table 4), and two haplotypes with 8 and 3 SNPs in the *ESRRG* gene (Table 5).

**Table 1:** Frequency, average effect, and respective standard error of haplotypes alleles located in the *PAPP-A2* gene.

*Alleles* *Haplotypes 16jc6*	*Bases of the SNPs*	*Frequency*	*Average effect*	*Standard error*
Allele 1	AGACCGCGTAAATGGTTTCA	0.527762	-18.0658	27.5808
**Allele 2**	**AAACAAAGTAGGCGTTCTCC**	**0.109384**	**-119.58**	**45.1731**
Allele 3	GAACAAAGTAGGCGTTCTCC	0.110772	55.0690	44.3902
Allele 4	AGACCGCAGGAACAGTTTCC	0.078845	-95.5179	51.3268
Allele 5	AGACCGCGTGGACGGTTTCC	0.063021	-78.7045	56.9129
Allele 6	AGACCGCAGGAACAGTTTCA	0.057746	49.6128	61.1468
**Allele 7**	**AGGTCGCAGGAACGGCTCTC**	**0.052471**	**207.18**	**59.5146**
**Haplotype 16kc3**				
Allele 1	CGAAT	0.582454	-19.2422	15.1954
Allele 2	AAGGC	0.222654	-27.9854	18.0778
**Allele 3**	**CGGAT**	**0.143254**	**-56.2382**	**20.7453**
**Allele 4**	**AAGAC**	**0.051638**	**103.47**	**29.6901**
**Haplotype 16lc3**				
Allele 1	GCCGG	0.616602	-16.5193	15.4988
Allele 2	GCTAG	0.228207	-14.7703	18.5367
**Allele 3**	**GTTAA**	**0.092171**	**-64.6040**	**24.6625**
**Allele 4**	**ACATG**	**0.063021**	**95.8936**	**28.8740**
**Haplotype 16vc4**				
Allele 1	TTGGTTCATAGGGGTTTC	0.495835647	16.5989	20.4811
Allele 2	TCGGTTCATAGGGGTTTC	0.242365353	31.8859	23.7671
Allele 3	CCAGTTCATAGGGGTTGC	0.165463631	35.6756	26.4293
Allele 4	CCAACCAGCCAAAAGGGT	0.068017768	42.6865	39.5794
**Allele 5**	**CCAGTTCGTAGGGGTTTC**	**0.028317601**	**-126.85**	**42.6491**

**Table 2:** Frequency, average effect, and respective standard error of haplotype alleles located in the *PAPP-A* gene.

*Alleles*	*Bases of the SNPs*	*Frequency*	*Average effect*	*Standard error*
**Haplotype8ra2**				
**Allele 1**	**TC**	**0.529983**	**22.2923**	**9.3300**
**Allele 2**	**CC**	**0.316491**	**28.2590**	**10.2994**
**Allele 3**	**CT**	**0.153526**	**-50.5513**	**12.8593**

**Table 3:** Frequency, average effect, and respective standard error of haplotype alleles located in the *XKR4* gene.

*Allele*	*Bases de SNPs*	*Frequency*	*Average effect*	*Standard error*
**Haplotype14u2**				
**Allele 1**	**GGCG**	**0.71266**	**-21.8432**	**11.2046**
**Allele 2**	**GGCA**	**0.217379**	**-44.5622**	**13.5822**
**Allele 3**	**ATAA**	**0.069961**	**66.4054**	**18.4249**

**Table 4:** Frequency, average effect, and respective standard error of the haplotype alleles located in the *MBL-1* gene.

*Alleles*	*Bases of the SNPs*	*Frequency*	*Average effect*	*Standard error*
***Haplotype*28c2**				
**Allele 1**	**AG**	**0.537757**	**27.9164**	**9.3271**
Allele 2	GA	0.25819	13.0536	10.7418
**Allele 3**	**GG**	**0.204053**	**-40.9700**	**11.7133**

**Table 5:** Frequency, average effect, and respective standard error of the haplotype alleles located in the *ESRRG* gene.

*Alleles*	*Bases of the SNPs*	*Frequency*	*Average effect*	*Standard error*
***haplotypes 16K5***				
Allele 1	GCGCATAA	0.401444	19.1973	0.4522
Allele 2	GCATATGG	0.188229	26.7919	0.3935
Allele 3	TTATGCGG	0.182121	-18.2323	0.5587
**Allele 4**	**GCATATAG**	**0.114381**	**82.8654**	**0.0252**
Allele 5	GTATATAG	0.082454	48.2178	0.2651
**Allele 6**	**GCGCATAG**	**0.031371**	**-158.84**	**0.0119**
**haplotypes 16pa2**				
**Allele 1**	**CGC**	**0.445586**	**48.2371**	**17.4130**
Allele 2	TAC	0.276791	33.0251	18.7878
Allele 3	TGT	0.245419	26.3177	19.3744
**Allele 4**	**TGC**	**0.032204**	**-107.58**	**38.7088**

## Discussion

The present results showed that the pre-selection of candidate genes and association analysis of SNPs that occur within these genes are efficient in identifying markers for sexual precocity in Nellore heifers.

The *ESRRG* (Estrogen-Related Receptor Gamma) gene was associated with sexual precocity in Nellore heifers. Members of the *ESRR* family (*ESRRα*, *ESRRβ* and *ESRRγ*) are orphan nuclear receptors and were identified based on their homology with estrogen receptors. Despite this similarity, *ESRRs* are not activated by estrogen [18]. *ESRRG* targets a variety of receptors such as follicle-stimulating hormone receptor (*FSHR*), GABA receptor (*GABRA1*) and glutamate receptor (*NMDAR2B*) etc. Through the ESRRG the FSHR may influence the feedback of estrogen on FSH release. The fact that ESRRG regulates GABRA1 and NMDAR2B indicates a link between estrogen pathways and GABA and glutamate signaling, which might influence puberty by activating the pulsatile secretion of GnRH [10]. Our findings corroborate the results of Fortes et al. [19] who performed genome-wide association studies (GWAS) to investigate the association between different genes and puberty in beef heifers. Puberty was defined as the age of occurrence of the first corpus luteum (AGECL). The authors observed a pleiotropic effect of the *ESRRG* gene in analyses of the gene network that considers AGECL as the main trait. Other studies conducted by the same group also associated the ESRRG gene with puberty in beef heifers [10] and [19].

Among reproductive tissues, studies have shown that the human placenta expresses large amounts of ESRRG [20, 18]. *ESRRG* participates in the formation of the syncytium and its expression increases during trophoblast differentiation. Since trophoblastic cells are essential for implantation of the embryo and development of the placenta during pregnancy in humans, mitochondrial biogenesis and the expression of some target genes of placental energy metabolism were found to be reduced when the expression of ESRRG was compromised [18]. The effect of the *ESRRG* gene on early heifer pregnancy identified in the present study may be due to its important role in metabolism and placental formation at the beginning of pregnancy.

Another pathway whereby *ESRRG* might interfere with early heifer pregnancy is through its action on lipid homeostasis and oxidative energy metabolism [21]. Functional genome studies have shown that *ESRR*s play an important role in the regulation of genes that encode components involved in pathways of fatty acid and glucose metabolism [22, 23, 18]. The relationship of a gene involved in lipid metabolism with a reproductive trait is the result of the fact that cycling females need to reach a certain body condition score, which is given in the final stage by the accumulation of fat, i.e., females are not reproductively active if they do not accumulate energy. A minimum body condition (i.e., total body fat) is therefore necessary for successful puberty. This is achieved when heifers reach 65% of the body weight of an adult cow to conceive at the beginning of the breeding season [24]. According to Semmelmann et al. [25], Nellore heifers that conceived after sexual maturation at 16 to 18 months of age were heavier and had a better body condition.

Two other genes that also exerted a significant effect (p<0.05) on sexual precocity in Nellore heifers were *PAPP-A* (pregnancy-associated plasma protein A) and *PAPP-A2* (pregnancy-associated plasma protein A2), which are metalloproteinases. These genes are located on chromosomes 8 and 16 and regulate the local bioavailability of insulin-like growth factor (*IGF*) in different systems including the placenta, ovarian follicles and bones and in pathological conditions such as skin wounds and atherosclerotic plaques [26]. *PAPP-A2* is a *PAPP-A* homolog that shares 46% sequence identity with the latter. This protein is a protease that specifically cleaves IGF-binding protein 5 (*IGFBP-5*) independent of IGF-1 [27] resulting in increased bioavailability of IGF, with significant consequences for the development of the fetus and the health of the mother [28].

In Holstein cows, a QTL influencing calving ease was detected on BTA16 in a region encompassing the *PAPP-A2* gene [29]. SNPs in these genes have also been associated with fertility traits, milk production and reproductive health in Holstein cattle [30]. Luna-Nevarez et al. [31] http://journals.plos.org/plosone/article?id=10.1371/journal.pone.0172330 - pone.0172330.ref002] associated SNPs in the *PAPP-A2* gene with reproductive traits in beef heifers and indicated this gene as a potential candidate for fertility in cattle, a fact that would explain the influence of the gene on sexual precocity in Nellore heifers.

In cattle *PAPP-A* cleaves both *IGFBP-4* and *IGFBP-5* in the presence of *IGF-1*. In the female reproductive system, *PAPP-A* is involved in the development of the ovarian follicle [32] and in the implantation of the embryo [33], a reduction in the expression of the *PAPP-A* gene is associated with the development of cysts in the ovary [34].

Furthermore, studies investigating bovine ovarian follicles demonstrated a strong association between IGF-1 concentrations and estradiol in follicular fluid and the capacity of these follicles to reach the preovulatory stage [35, 36]. *PAPP-A* may play a role in ovarian follicles, participating especially in oocyte maturation, irrespective of the cleavage of *IGFBP-4*. FSH was found to stimulate the expression of the IGFR-1 and PAPP-A genes in granulosa cells, suggesting that the PAPP-A mRNA expression in granulosa cells may contribute to the production of free IGF-I and increase LH receptors to trigger the preovulatory phase and consequently lead to ovulation [37, 38]. Finally, it is possible that *PAPP-A* participates in ovulation through the cleavage of critical substrates [39, 38]. According to Fortune et al. [40], the FSH-induced increase in *PAPP-A* is the earliest change detected in the future dominant follicle. This would explain the role of *PAPP-A* in sexual precocity since this gene is involved in ovulation and puberty. From a hormone point of view, puberty is defined as the first sign of estrous behavior associated with a potentially fertile ovulation, followed by a corpus luteum of normal duration [41].

Taken together, all of these reports show a strong association of sexual precocity with the *PAPP-A* and *PAPP-A2* genes via *IGF-1*. Although no significant effect of the *IGF-1* gene was observed in the present study, this gene plays a key role in the determination of age at puberty. This is the fact that IGF-1 acts in the follicles promoting an increase in estradiol production, thus increasing steroidogenesis [42] and IGF-I encourage puberty by acting on the central nervous system (mean eminence), leading to an increase in the release of GnRH, which would stimulate the increase in the frequency of LH pulses [43, 44].

A significant difference in sexual precocity of heifers was observed for the *XKR4* gene (XK, Kell blood group complex subunit-related family, member 4). This gene is expressed preferentially in the cerebellum [45, 46]. The amino acid sequence of the *XKR4* gene has important biological functions in cell and lipid metabolism [47]. Utsunomiya et al. [48] performed GWAS to identify chromosome regions that affect birth weight in Nellore cattle. The authors found a significant SNP (rs42646720) in intron 2 of the *XKR4* gene and reported its association with birth weight and size in Zebu breeds (*Bos primigenius indicus*).

Lindholm-Perry et al. [47] identified five SNPs located between the TMEM68 and *XKR4* genes that were significant for residual feed intake and average daily gain in crossbred steers. Bolormaa et al. [49] found five SNPs in a region of BTA14 encompassing *XKR4* that were associated with rump fat thickness at position P8 (CHILLP8) in seven cattle breeds. In another study investigating the *XKR4* gene in cattle, three SNPs associated with rump fat thickness were detected within the gene [50]. The *XKR4* gene has been described as a potential candidate gene associated with residual feed intake, average daily feed intake, average daily gain and fat accumulation. This would explain its relationship with sexual precocity since the onset of puberty and sexual maturity in female cattle is influenced, among many factors, by the rate of weight gain and body condition [51].

The mannose-binding lectin (*MBL*) gene also influenced sexual precocity in Nellore heifers. This lectin is found in human serum, is secreted by the liver, and binds carbohydrates in a calcium-dependent manner. *MBL* is an important element of the innate immune system that is involved in the activation of the complement system via lectins and functions as an opsonin, facilitating the phagocytosis of pathogens. Serum MBL deficiency is associated with infectious and autoimmune diseases, interfering with both the susceptibility to and severity of the disease [52, 53, 54].

Serum concentrations of *MBL* increase significantly from the first trimester of gestation and decrease 6 weeks after delivery. *MBL* contributes to normal placentation during an ongoing pregnancy. The reasons for increased *MBL* levels during pregnancy are unknown. It has been suggested that adaptive immunity decreases during pregnancy, which allows the pregnant body to tolerate the fetus as an allograft [55, 56].

Low *MBL* concentrations have been associated with spontaneous abortions [57], suggesting that an altered immune response in the fetal environment is responsible for the increased susceptibility to abortion. This could explain the function of this gene in sexual precocity. Precocious heifers would have a more competent immune system and are consequently more likely to conceive early and to carry the pregnancy to term since a successful pregnancy depends on the tolerance of the maternal immune system to a new organism with its own characteristics that differ from those of the mother. If this immune adaptation does not occur, the embryo is destroyed and expelled [58, 59].

Cochran et al. [9] studied genes associated with reproductive and other traits and found seven genes with SNPs associated with pregnancy rate that are involved in immune function. Hansen et al. [60] emphasized the importance of the immune system for the establishment of pregnancy in mammals.

Another function of *MBL-1* is related to lipid metabolism in humans. Rakhshandehroo et al. [61] concluded that the PPARα stimulates plasma levels and gene expression of *MBL* in hepatocytes, suggesting a possible role of MBL in lipid metabolism. This finding could explain the influence of the MBL gene on sexual precocity. In Nellore heifers, Dias et al. [62] observed that the onset of puberty was influenced by two genes related to lipid metabolism. Some factors such as age and body fat reserves can affect the onset of the estrous cycle. The increase in body fat deposition reduces the negative feedback caused by estrogens on GnRH and LH pulsatility, with adequate energy intake being the main nutritional factor that affects the reproductive performance of cows [63]. Heifers with a positive diagnosis of pregnancy at 13–15 months of age are heavier and older and have a better body condition than non-pregnant heifers [64].

It should be noted that the *ESRRG* and *XKR4* genes can also influence lipid metabolism as reported earlier, demonstrating the importance of these genes for sexual precocity in Nellore heifers since genes that affect fat deposition and, consequently, body condition play an important role in Zebu breeding.

## Conclusion

The *PAPP-A2*, *PAPP-A*, *ESRRG*, *MBL-1* and *XKR4* genes, which participate in specific events in target tissues of cattle reproduction (hypothalamic-pituitary-gonadal axis, adipose tissue and immune system), were associated with sexual precocity in Nellore heifers and could help identify the main physiological factors that contribute to expression of the phenotype. In addition, these genes are candidates for prospecting causal mutations in future studies and are potential genes to be included in genetic evaluations.

## Supporting information

S1 File File with results from the first part of the statistical analyzes. (ZIP)

S2 File Files with results from the second part of the statistical analyzes. (XLS)

S3 File File with the genotypes of all animals used in this study. (RAR)

S1 Table List of candidate genes, description, chromosome and number of SNPs found in each gene. (DOCX)

S2 Table Haplotype location and position of the chromosomes and SNPs of genes with a significant effect at p<0.05. (DOCX)
