# Supplementary material for: Genomic association for sexual precocity in beef heifers using pre-selection of genes and haplotype reconstruction
Source: PLoS One. 2018 Jan 2;13(1):e0190197. doi: 10.1371/journal.pone.0190197 (PMC5749767; doi:10.1371/journal.pone.0190197)
Supplement: S1 Table — (DOCX) [file pone.0190197.s004.docx]

**S1 Table.** List of candidate genes, description, chromosome and number of SNPs found in each gene.

| Gene | **Descripton Chromosome** | |  | **No. of SNPs** |
| --- | --- | --- | --- | --- |
| *ACVR* | Activin | 2 | | 18 |
| *AREG* | Amphiregulin B | 6 | | 8 |
| *3BDNF* | Brain derived neurotrophic factor | 15 | | 8 |
| *BTC* | Betacellulin | 6 | | 22 |
| *BMP2* | Bone morphogenetic protein 2 | 13 | | 3 |
| *BMP4* | Bone morphogenetic protein 4 | 10 | | 0 |
| *BMP6* | Bone morphogenetic protein 6 | 23 | | 52 |
| *BMP7* | Bone morphogenetic protein 7 | 13 | | 29 |
| *BMP15* | Bone morphogenetic protein 15 | X | | 0 |
| *BMPIA* | Receptor BMP tipo IA | 28 | | 8 |
| *BMPIB* | Receptor BMP tipo IB | 6 | | 6 |
| *CAMTA 1* | Calmodulin binding transcription activator 1 | 16 | | 13 |
| *c-Kit* | KIT proto-oncogene receptor tyrosine kinase | 6 | | 24 |
| *CNOT2* | CCR4-NOT transcription complex, subunit 2 | 5 | | 10 |
| *CRH* | Corticotropin releasing hormone | 14 | | 3 |
| *CYP* | Cytochrome P450 | 10 | | 6 |
| *DRD3* | Dopamine receptor | 10 | | 1 |
| *EGF* | Epidermal growth factor | 6 | | 35 |
| *EGFR* | Epidermal growth factor receptor | **22** | | 35 |
| *EPHA4* | EPH receptor A4 | 2 | | 17 |
| *EPR* | Epiregulin | 6 | | 4 |
| *ErbB* | ERBB receptor feedback inhibitor 1 | 16 | | 6 |
| *ERα* | Estrogen receptor alfa | 9 | | 4 |
| *ERβ* | Estrogen receptor beta | 10 | | 9 |
| *ESRRG* | Estrogen-related receptor gamma | 16 | | 199 |
| *EYA1* | Eyes absent homolog 1 | 14 | | 76 |
| *FGF-1* | Fibroblast growth factor 1 | 7 | | 12 |
| *FGF-2* | Fibroblast growth factor 2 | 17 | | 16 |
| *FGF-7* | Fibroblast growth factor 7 | 10 | | 5 |
| *FGF-8* | Fibroblast growth factor 8 | 26 | | 1 |
| *FGFR-2* | Fibroblast growth factor receptor 2 | 26 | | 12 |
| *FGFR-3* | Fibroblast growth factor receptor 3 | 6 | | 3 |
| *Flk-1* | Kinase insert domain receptor | 6 | | 15 |
| *FLT-1* | Vascular endothelial growth fator 1 | 12 | | 42 |
| *FSHR* | Follicle stimulating hormone receptor | 11 | | 90 |
| *FST* | Follistatin | 20 | | 3 |
| *GABARAP* | GABA type A receptor-associated protein | 19 | | 0 |
| *GABRA* | Gamma-aminobutyric acid type a receptor alpha1 subunit | 7 | | 14 |
| *Gata 4* | GATA binding protein 4 | 8 | | 16 |
| *GHR* | Growth hormone receptor | 20 | | 42 |
| *GHRH* | Growth hormone releasing hormone | 13 | | 2 |
| *GnRHR* | Gonadotropin-releasing hormone receptor | 6 | | 8 |
| *GPR54* | KiSS-1 receptor-like | 7 | | 3 |
| *HB-EGF* | Heparin-binding EGF-like growth factor | 7 | | 0 |
| *H1Foo* | H1 histone family, member O, oocyte-specific | 22 | | 11 |
| *H2A* | Histone H2A | 23 | | 3 |
| *HIVEP3* | Human immunodeficiency virus type I enhancer binding protein 3 | 3 | | 15 |
| *17β-HSD* | Hydroxysteroid (17-beta) dehydrogenase | 5 | | 2 |
| *IFNT2* | Interferon tau | 8 | | 1 |
| *IGF-1* | Insulin-like growth factor 1 | 5 | | 3 |
| *IGF-2* | Insulin-like growth factor 2 | 29 | | 1 |
| *IGFBP-1* | Insulin-like growth factor binding protein1 | 4 | | 1 |
| *IGFBP-2* | Insulin-like growth factor binding protein 2 | 2 | | 15 |
| *IGFBP-3* | Insulin-like growth factor binding protein3 | 4 | | 5 |
| *IGFBP-4* | Insulin-like growth factor binding protein 4 | 19 | | 1 |
| *IGFBP-5* | Insulin-like growth factor binding protein 5 | 2 | | 11 |
| *IGFBP-6* | Insulin-like growth factor binding protein 6 | 5 | | 3 |
| *IGFR-1* | Insulin-like growth factor 1 receptor | 21 | | 61 |
| *IGFR-2* | Insulin-like growth fator 2 receptor | 9 | | 20 |
| *IHH* | Indian hedgehog | 2 | | 2 |
| *IL-1A* | Interleucin 1A | 11 | | 1 |
| *IL-6* | Interleucin 6 | 4 | | 4 |
| *IL-10* | Interleucin 10 | 16 | | 0 |
| *INHA* | Inhibin alpha subunit | 2 | | 2 |
| *Kiss-1* | KiSS-1 metastasis-suppressor | 16 | | 5 |
| *KL* | Kit Ligant | 5 | | 12 |
| *LHR* | Luteinizing hormone receptor | 11 | | 19 |
| *LIF* | Leukemia inhibitory factor | 17 | | 3 |
| *MBL-1* | Mannose Binding Lectin | 28 | | 7 |
| *MC2R* | Melanocortin 2 receptor | 24 | | 4 |
| *MOS* | V-mos Moloney murine sarcoma viral oncogene homolog | 14 | | 3 |
| *MTNR1A* | Melatonin receptor 1A | 27 | | 2 |
| *NCOA2* | Nuclear receptor coactivator 2 | 14 | | 38 |
| *NELL2* | NEL-like 2 | 5 | | 102 |
| *NGF* | Nerve growth factor | 3 | | 6 |
| *NGFR* | Nerve growth factor receptor | 19 | | 5 |
| *NMDA* | Glutamate receptor | 5 | | 49 |
| *NRG1* | Neuregulin 1 | 27 | | 55 |
| *NR6A1* | Nuclear receptor subfamily 6, group A, member 1 | 11 | | 6 |
| *NTF-3* | Neurotrophin 3 | 5 | | 20 |
| *NTF4* | Neurotrophin 4 | 18 | | 0 |
| *NYP* | Neuropeptide Y | 4 | | 1 |
| *OB-Rs* | Leptin receptor | 3 | | 6 |
| *OOSP1* | Oocyte-secreted protein 1 | 15 | | 3 |
| *OXT* | Oxytocin | 13 | | 1 |
| *PAPP-A* | Pregnancy-associated plasma protein A | 8 | | 108 |
| *PAPP-A2* | Pregnancy-associated plasma protein A2 | 16 | | 91 |
| *PENK* | Proenkephalin | 14 | | 5 |
| *PGR* | Progesterone receptor | **1**5 | | 3 |
| *PGF2R* | Prostaglandin F receptor | 3 | | 15 |
| *PLAG1* | Pleiomorphic adenoma gene 1 | 14 | | 5 |
| *PLAGL1* | Pleiomorphic adenoma gene-like 1 | 9 | | 3 |
| *POU3F4* | POU class 3 homeobox 4 | X | | 0 |
| *POMC* | Proopiomelanocortin | 11 | | 7 |
| *PPARG* | Peroxisome proliferator-activated receptor gamma | 22 | | 17 |
| *PRL* | Prolactin | 23 | | 7 |
| *PRLR* | Prolactin receptor | 20 | | 13 |
| *PROP1* | PROP Paired-like homeobox 1 | 7 | | 4 |
| *PTX3* | Pentraxin 3, long | 1 | | 4 |
| *RFX4* | Regulatory factor X, 4 | 5 | | 8 |
| *RPS20* | Ribosomal protein S20 | 14 | | 3 |
| *SCMH1* | Sex comb on midleg homolog 1 | 3 | | 17 |
| *SERPINA 7* | Serpin peptidase inhibitor, member 7 | X | | 0 |
| *SF-1* | Nuclear receptor subfamily 5, group A, member 1 | 11 | | 7 |
| *SLBP* | Stem loop bindimg protein | 6 | | 2 |
| *SLC6A2* | Noradrenalin | 18 | | 14 |
| *SNAI2* | Snail homolog 2 | 14 | | 3 |
| *STAR* | Steroidogenic acute regulatory protein | 27 | | 2 |
| *STAT2* | Signal transducer and activator of transcription 2 | 5 | | 6 |
| *STAT6* | Signal transducer and activator of transcription 6 | 5 | | 0 |
| *STK36* | Serine/threonine kinase 36 | 2 | | 8 |
| *TAF-1* | RNA polymerase II, TATA box binding protein (TBP)-associated fator 1 | X | | 0 |
| *TAF9B* | RNA polymerase II, TATA box binding protein (TBP)-associated factor | X | | 0 |
| *TGFα* | Transforming growth factor alpha | 11 | | 13 |
| *TGFβ* | Transforming growth factor beta | 18 | | 4 |
| *TOX* | Thymocyte selection-associated high mobility group box | 14 | | 73 |
| *TTF-1* | Transcription termination fator 1 | 11 | | 19 |
| *VEGFA* | Vascular endothelial growth factor A | 23 | | 2 |
| *VEDFB* | Vascular endothelial growth factor B | 29 | | 1 |
| *VIP* | Vasoactive intestinal peptide | 9 | | 5 |
| *WNT6* | Wingless-type MMTV integration site Family member 6 | 2 | | 1 |
| *XKR4* | Kell blood group complex subunit-related Family member 4 | 14 | | 85 |
| *ZFHX4* | Zinc finger homeobox 4 | 14 | | 49 |
| *ZMAT3* | Zinc finger matrin-type 3 | 1 | | 3 |
| *ZNF462* | Zinc finger protein 462 | 8 | | 28 |
