# Supplementary material for: Genomic association for sexual precocity in beef heifers using pre-selection of genes and haplotype reconstruction
Source: PLoS One. 2018 Jan 2;13(1):e0190197. doi: 10.1371/journal.pone.0190197 (PMC5749767; doi:10.1371/journal.pone.0190197)
Supplement: S2 Table — (DOCX) [file pone.0190197.s005.docx]

**S2 Table.** Haplotype location and position of the chromosomes and SNPs of genes with a significant effect at p<0.05.

| LocalizationSNP_Name | Chr | Position |
| --- | --- | --- |
| **ALE16K gene *ESRRG*** |  |  |
| BovineHD1600005722 | 16 | 20644687 |
| BovineHD1600005723 | 16 | 20645666 |
| BovineHD1600005724 | 16 | 20646546 |
| BovineHD1600005725 | 16 | 20648391 |
| BovineHD1600005728 | 16 | 20656102 |
| BovineHD1600005729 | 16 | 20660985 |
| ARS-BFGL-BAC-4819 | 16 | 20663521 |
| BovineHD1600005730 | 16 | 20664151 |
| **ALE16PA Gene *ESRRG*** |  |  |
| BovineHD1600005818 | 16 | 20897289 |
| Hapmap25031-BTA-130 | 16 | 20908000 |
| BovineHD1600005821 | 16 | 20914819 |
| **ALE16JC gene *PAPPA-A2*** |  |  |
| BovineHD1600016515 | 16 | 59140875 |
| BovineHD1600016516 | 16 | 59143533 |
| BovineHD1600016517 | 16 | 59144080 |
| BovineHD1600016518 | 16 | 59147686 |
| BTB-00583289 | 16 | 59154339 |
| BovineHD1600016519 | 16 | 59159178 |
| BovineHD1600016520 | 16 | 59164615 |
| BovineHD1600016521 | 16 | 59166341 |
| BovineHD1600016523 | 16 | 59172092 |
| BovineHD1600016524 | 16 | 59172656 |
| BovineHD1600016525 | 16 | 59173651 |
| BovineHD1600016526 | 16 | 59178278 |
| BovineHD1600016527 | 16 | 59180192 |
| BovineHD1600016528 | 16 | 59181746 |
| BovineHD1600016529 | 16 | 59182205 |
| BovineHD1600016530 | 16 | 59183170 |
| BovineHD1600016531 | 16 | 59186597 |
| Hapmap41703-BTA-979 | 16 | 59188330 |
| BovineHD1600016532 | 16 | 59189571 |
| BovineHD1600016533 | 16 | 59193302 |
| BovineHD1600016534 | 16 | 59193783 |
| **ALE16KC gene *PAPP-A2*** |  |  |
| BovineHD1600016535 | 16 | 59196392 |
| BovineHD1600016536 | 16 | 59201399 |
| BovineHD1600016537 | 16 | 59205592 |
| BovineHD1600016538 | 16 | 59208865 |
| BovineHD1600016540 | 16 | 59215453 |
| **ALE16LC gene *PAPP-A2*** |  |  |
| BovineHD1600016543 | 16 | 59234237 |
| BovineHD1600016544 | 16 | 59240208 |
| BovineHD1600024596 | 16 | 59242735 |
| BovineHD1600016545 | 16 | 59251366 |
| BovineHD1600016546 | 16 | 59254062 |
| **ALE16VC Gene *PAPP-A2*** |  |  |
| BovineHD1600024598 | 16 | 59374204 |
| BovineHD1600016592 | 16 | 59377093 |
| BovineHD1600016593 | 16 | 59383104 |
| BovineHD1600016594 | 16 | 59387369 |
| BovineHD1600016596 | 16 | 59394776 |
| BovineHD1600016597 | 16 | 59397401 |
| BovineHD1600016598 | 16 | 59401319 |
| BovineHD1600016599 | 16 | 59408840 |
| BovineHD1600016601 | 16 | 59414846 |
| BovineHD1600016602 | 16 | 59430795 |
| BovineHD1600016603 | 16 | 59431977 |
| BovineHD1600016604 | 16 | 59432785 |
| BovineHD1600016607 | 16 | 59438692 |
| BovineHD1600016608 | 16 | 59439619 |
| BovineHD1600016609 | 16 | 59441774 |
| BovineHD1600016612 | 16 | 59444184 |
| BovineHD1600016614 | 16 | 59446488 |
| BovineHD1600016615 | 16 | 59449513 |
| BovineHD1600016616 | 16 | 59452291 |
| **ALE8RA Gene *PAPP-A*** |  |  |
| BovineHD0800032050 | 8 | 107173877 |
| BovineHD0800032051 | 8 | 107177141 |
| **AL14U Gene *XKR4*** |  |  |
| BTB-01530778 | 14 | 24482969 |
| BovineHD1400007109 | 14 | 24485000 |
| BovineHD1400007111 | 14 | 24491669 |
| BovineHD1400007112 | 14 | 24493787 |
| **ALE28C gene *MBL-1*** |  |  |
| BovineHD2800009737 | 28 | 35848678 |
| BovineHD2800009738 | 28 | 35849471 |
